# Comparison of white blood and black blood T2* cardiovascular magnetic resonance at 3 Tesla

**DOI:** 10.1186/1532-429X-16-S1-O86

**Published:** 2014-01-16

**Authors:** Mohammed H Alam, Gillian C Smith, John Paul Carpenter, Arun J Baksi, Ricardo Wage, Peter Drivas, Karen Symmonds, Taigang He, David Firmin, Dudley J Pennell

**Affiliations:** 1 NIHR Cardiovascular Biomedical Research Unit, Royal Brompton Hospital, London, UK; 2 Imperial College London, London, UK

## Background

T2* relaxometry offers a non-invasive method to accurately quantify myocardial iron levels, thus aiding the diagnosis and assessment of prognosis of patients. Two T2* sequences are used clinically: the single breath-hold multi-echo gradient echo white blood (WB) and black blood (BB) techniques. At 1.5T, BB T2* has superior reproducibility and fewer artefacts than WB T2*. We compared BB and WB T2* iron measurement at 3T and compared results with corresponding 1.5T measurements.

## Methods

We recruited 100 patients (55 male, aged 14 to 81 years) referred for iron assessment (thalassemia major 43, sickle cell disease 15, hereditary haemochromatosis 11, other iron overload conditions 31). The same mid-ventricular short axis slices were used to generate both BB and WB T2* images at 1.5T (Avanto, Siemens, Erlangen, Germany), and similarly at 3T (Skyra). 20 patients underwent repeat studies on the same day for reproducibility. Septal regions of interest were analyzed using CMRtools, using the truncation algorithm. All patient images were ranked for artefact severity on an ordinal scale from 0 (uninterpretable images) to 5 (no septal artefact).

## Results

**Figure 1:**
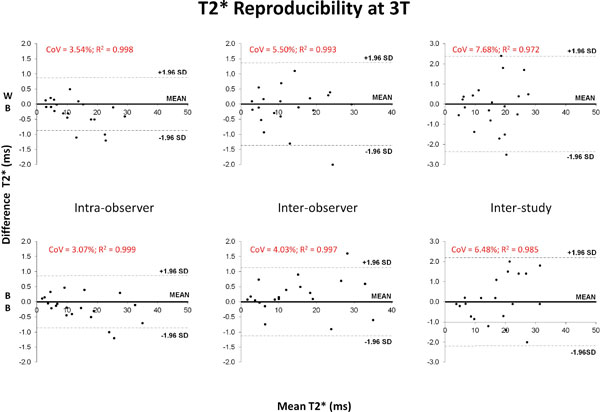
Bland Altman plots comparing myocardial white blood and black blood T2* reproducibility at 3T.

**Figure 2:**
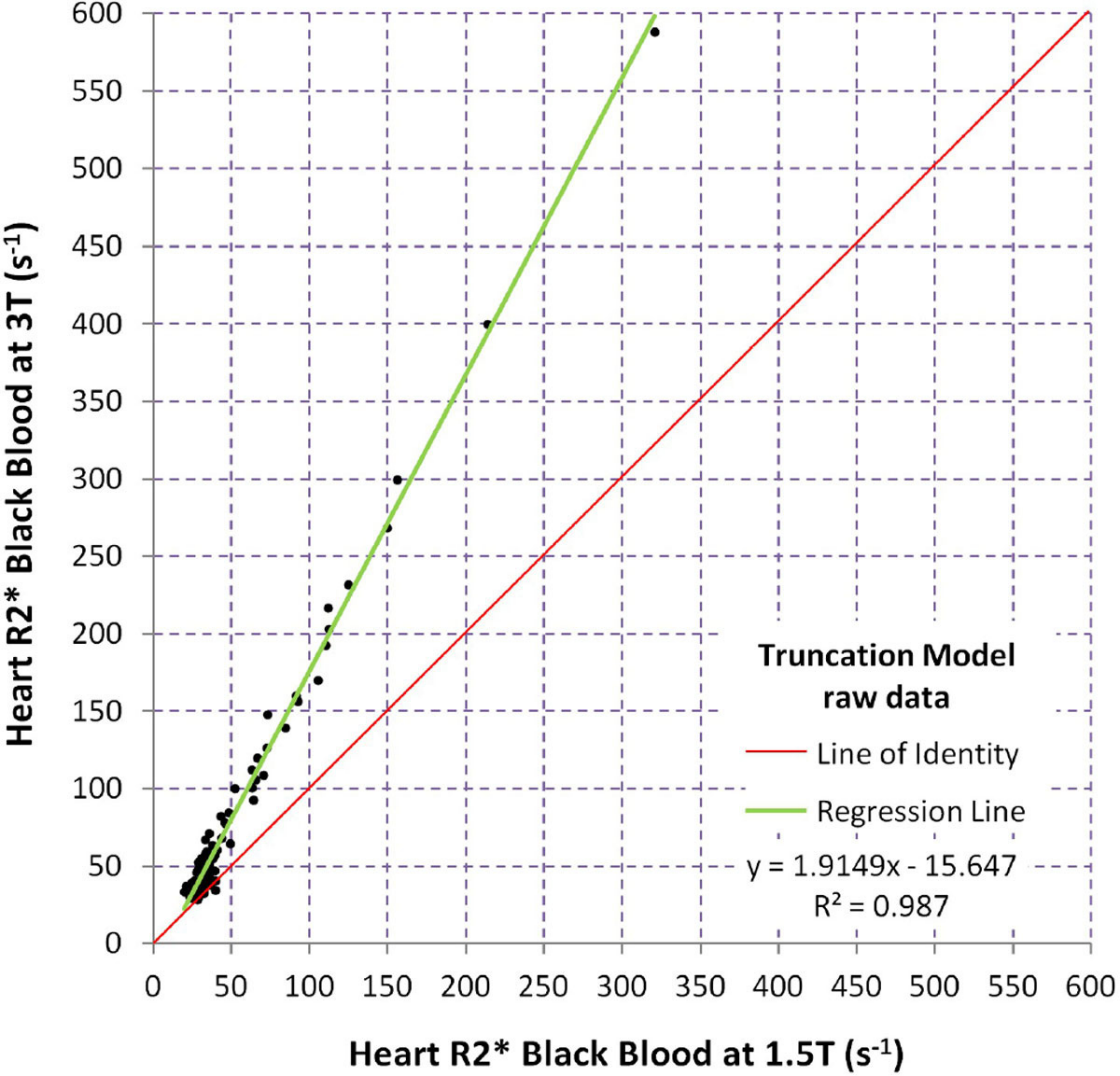
Scatter plot showing correlation of black blood myocardial T2* values at 3T with corresponding 1.5T values.

Median BB T2* was 29.8 ms (range 3.12-50.5 ms) at 1.5T, and 21.4 ms (range 1.70-35.5 ms) at 3T. Median WB T2* values were 30.8 ms (range 3.76-49.2 ms) at 1.5T and 19.0 ms (range 2.82-34.7 ms) at 3T. Intra-observer, inter-observer and inter-study reproducibility measurements at 3T of BB T2* yielded coefficients of variation (CoV) of 3.1%, 4.0% and 6.5% respectively, which were superior to WB T2* whose CoV were 3.5%, 5.5% and 7.7% respectively (Figure 1). Mean artefact score was 4.11 for BB T2* at 3T, which was significantly better than WB T2* at 3T (3.45; p < 0.001). Applying the truncation algorithm to both 1.5T and 3T analyses, BB R2* (1000/T2*) correlated more strongly to 1.5T values (slope 1.91, intercept -15.6, Rsquared = 0.987; Figure 2) than WB R2* (slope 1.61, intercept +2.96, Rsquared = 0.947).

## Conclusions

At 3T, BB T2* is superior to WB T2* for artefacts, noise and reproducibility, which accords with experience at 1.5T. The 3T BB T2* values correlate well with gold standard 1.5T T2* with values approximately halved. Considerable further experience with BB T2* is needed before robust clinical practice can be recommended

## Funding

This research was supported by the NIHR Cardiovascular Biomedical Research Unit at Royal Brompton & Harefield NHS Foundation Trust and Imperial College London.

